# Immunoglobulin Y-Based Lateral Flow Immunoassay Strip Test for Detecting Ciprofloxacin Antibiotic in Raw Pork Samples

**DOI:** 10.3390/foods14050818

**Published:** 2025-02-27

**Authors:** Sumed Yadoung, Huan-Yuan Xu, Sirikwan Dokuta, Peerapong Jeeno, Pichamon Yana, Marninphan Thongkham, Korawan Sringarm, Ryoichi Ishimatsu, Zhen-Lin Xu, Surat Hongsibsong

**Affiliations:** 1 Environmental Science Program, Faculty of Science, Chiang Mai University, Chiang Mai 50200, Thailand; sumed_y@cmu.ac.th; 2 Environmental, Occupational Health Sciences and Non-Communicable Diseases Center of Excellence, Research Institute for Health Sciences, Chiang Mai University, Chiang Mai 50200, Thailand; pichamon.y@cmu.ac.th; 3 Guangdong Provincial Key Laboratory of Food Quality and Safety, South China Agricultural University, Guangzhou 510642, China; xhy578442745@163.com; 4 School of Health Sciences Research, Research Institute for Health Sciences, Chiang Mai University, Chiang Mai 50200, Thailand; sirikwan_d@cmu.ac.th (S.D.); peerapong_jeen@cmu.ac.th (P.J.); 5 Office of the University, Chiang Mai University, 239 Huay Keaw Road, Suthep, Muang, Chiang Mai 50200, Thailand; 6 Department of Animal and Aquatic Sciences, Faculty of Agriculture, Chiang Mai University, Chiang Mai 50200, Thailand; marninphan_t@cmu.ac.th (M.T.); korawan.s@cmu.ac.th (K.S.); 7 Department of Applied Physics, University of Fukui, 3-9-1 Bunkyo, Fukui 910-8507, Japan; ishima2@u-fukui.ac.jp

**Keywords:** ciprofloxacin, enrofloxacin, immunoglobulin Y-based, IgY antibody, lateral flow immunoassay strip test, polyclonal

## Abstract

Ciprofloxacin is metabolized from enrofloxacin for use in poultry to manage respiratory and gastrointestinal diseases, raising concerns due to its widespread tissue distribution and prolonged systemic persistence. This lateral flow immunoassay was designed to detect ciprofloxacin using an alternative IgY antibody binded with gold nanoparticles to detect ciprofloxacin residue in raw pork meat samples. The developed strip test achieved adequate sensitivity and specificity under the optimized conditions for pH, which is 7.8, and 20% of MeOH in 0.01 M phosphate buffer containing 1% Tween-20 was used for the buffer composition. An antibody concentration of 1.25 µg/mL was used to bind with gold nanoparticles as a probe for detection. The concentration of the test line (coating antigen) and control line (anti-IgY secondary antibody) was 0.5 mg/mL and 0.2 mg/mL, respectively. The efficiency of the developed strip test showed sensitivity with a 50% inhibitory concentration (IC_50_) of ciprofloxacin at 7.36 µg/mL, and the limit of detection was 0.2 µg/mL. The proposed strategy exhibited potential for monitoring ciprofloxacin in raw pork samples.

## 1. Introduction

Fluoroquinolone antibiotics (FQs), such as ciprofloxacin, are widely used in humans to treat respiratory, urinary, and gastrointestinal infections. However, humans may also be exposed to ciprofloxacin through daily meat consumption. Enrofloxacin, another fluoroquinolone antibiotic, is commonly used in poultry to manage respiratory and gastrointestinal diseases. Once administered, enrofloxacin can metabolize into ciprofloxacin, raising significant concerns due to its widespread tissue distribution and prolonged systemic persistence. These factors complicate adherence to the recommended withdrawal periods required to ensure the safety of poultry meat for consumption. This issue is particularly problematic as the legal withdrawal period often conflicts with the typical disease onset period in poultry. Additionally, enrofloxacin is frequently used on farms to produce meat, especially from pigs. Ciprofloxacin is tailored for human infections, while enrofloxacin is designed and approved for animal use. Their relationship is that enrofloxacin metabolizes into ciprofloxacin in animals, contributing to its effect. Regulatory and safety concerns dictate their respective uses. Ciprofloxacin residues in animal products raise concerns about indirect human exposure, which may contribute to antimicrobial resistance and allergic reactions [1].

Several studies have reported ciprofloxacin residues in pork samples. In India, 49 samples (6.8%) of 720 pork samples were found to contain ciprofloxacin residues [2]. In Nigeria, 40% of 80 pork samples contained ciprofloxacin, with mean concentrations of 315.30 μg/kg [3]. Similarly, in Thailand, 13.95% of 43 raw pork samples from local markets showed ciprofloxacin residues, along with antibiotic-resistant organisms, such as *Escherichia coli* and *Klebsiella pneumoniae* [4]. These findings reveal considerable variation in ciprofloxacin residue levels across countries. However, distinguishing ciprofloxacin originating from enrofloxacin metabolism from ciprofloxacin directly from human medical use remains a significant challenge. The statutory withdrawal period for veterinary medicinal products must not be less than 28 days for meat from poultry and mammals, as per European health laws and the National Office of Animal Health in the United Kingdom [5].

Consequently, ciprofloxacin residues may remain in the meat people ingest, potentially resulting in antibiotic resistance. The excessive residue of ciprofloxacin in pork products, together with resistance mechanisms such as diminished drug permeability and bacterial mutation, presents a public health threat by decreasing the efficacy of this crucial antibiotic in treating human illnesses.

To ensure the safety of food and environmental products, researchers have developed and implemented a variety of detection methods for the analysis of environmental samples and food products. HPLC is the most frequently employed method, while LC-MS is the most versatile and widely used method due to its high sensitivity and ability to analyze multiple compounds [6]. Moreover, alternative reversal detection methods, such as optical detection methods (fluorescence detection), are used to detect FQs, which provide high sensitivity for specific compounds but may require additional steps for derivatization and electrochemical detection, which is useful for portable and on-site analysis but may face challenges with interference and requires careful sensor development. Also, immunoassay methods (enzyme-linked immunosorbent assay (ELISA) and lateral flow assays (LFA)) offer rapid and specific detection [7,8].

Immunoglobulin Y (IgY) is a kind of immunoglobulin that serves as the predominant antibody in the blood of birds, reptiles, and lungfish. It is also present at elevated concentrations in chicken egg yolks. IgY, like other immunoglobulins, is a class of proteins produced by the immune system in response to certain foreign chemicals, enabling their recognition. IgY is physically and functionally distinct from mammalian IgG, and it does not exhibit cross-reactivity with antibodies generated against mammalian IgG. IgY is simple to manufacture and economical. Egg-laying chickens can efficiently produce IgY antibodies in substantial volumes with minimal ecological impact or infrastructural investment [9].

The current study developed an immunoassay using the IgY antibody from immunized chickens with immunogen-stimulated hen egg yolks specific to ciprofloxacin antibiotics. The lateral flow immunoassay was designed to detect ciprofloxacin using IgY antibody binding with gold nanoparticles to detect residue in raw pork meat samples.

## 2. Materials and Methods

### 2.1. Chemicals and Materials

N-hydroxysuccinimide (NHS; AR grade, 99%), 1-Ethyl-3-(3-dimethylaminopropyl) carbodiimide (EDC, AR grade, 99.9%), chloroauric acid (HAuCl_4,_ AR grade, 98%), sucrose (C_12_H_22_O_11_, AR grade, 99%), ciprofloxacin (Cip, AR grade, 97%), and sodium azide (NaN_3_, AR grade, 99.9%) was purchased from Sigma-Aldrich, St. Louis, MO, USA. Lactoferrin (LF, AR grade, 98%) was obtained from Sigma-Aldrich, Darmstadt, Germany. Polyvinylpyrrolidone (PVP, AR grade, 99%) and polyethylene glycol 6000 (PEG 6000, AR grade, 98%) was purchased from Bio Basic, Markham ON, Canada. Polysorbate 20 or Tween-20 (AR grade, 99%) was obtained from Loba Chemie, Mumbai, India.

### 2.2. Preparation of Antigen for Control Line

The ciprofloxacin was conjugated to LF by a modified previously published method [10]. We mixed 11.5 mg EDC, 19.1 mg NHS, and 10 mg ciprofloxacin dissolved in 2.0 mL of carbonate buffer pH 9.6 and stirred for 2 h at room temperature. Moreover, 5.0 mL of 10 mg/mL LF in 50 mM carbonate buffer pH 9.6 was added to the solution. The mixture was allowed to react at room temperature for 4 h with stirring.

### 2.3. Production of IgY pAb Against Ciprofloxacin

The antibody was produced as described by Yadoung et al. [11] as applied to immunization in chicken hens. The collection of egg yolk occurred after boosted 4th onward.

### 2.4. Gold Nanoparticles Solution

The gold nanoparticles (AuNPs) were produced through the reduction of HAuCl_4_ using sodium citrate according to previously established methods [12]. Add 100 mL of ultra-pure water into a round-bottled flask. Subsequently, the flask should be placed in a magnetic stirring bath set at 180 °C with a stirring rate of 700 revolutions per minute. Initiate stirring and subject the contents to reflux until boiling occurs. Introduce 4 mL of a 1% chlorauric acid solution and continue heating and stirring persistently until boiling resumes. Promptly supplement 4.6 mL of a 1% trisodium citrate solution. Observe the rapid transition of the solution from yellow to colorless and transparent, followed by a subsequent transformation from transparent to black within one minute. Sustain continuous stirring and heating until a vivid wine-red hue emerges within 2 to 3 min. Cease heating after 10 min of uninterrupted application and maintain stirring under residual heat. Allow the solution to cool to room temperature before transferring it to a pristine glass bottle. Store the prepared solution at 4 °C for subsequent use.

### 2.5. Preparation of AuNPs-Labeled IgY pAb

The probe consisting of AuNPs combined with IgY antibody was produced utilizing the process of electrostatic adsorption. A suitable pH of conjugated condition was adjusted with 1% K_2_CO_3_ into the AuNPs solution, and antibody solution (5 µg/mL) was quickly added to pH adjusted AuNPs solution. Combination of the mixture of AuNPs and antibody for 5 min with vortex mixture at room temperature. Then, 10 μL of 10% BSA in distilled water was added and incubated for 1 min to prevent additional binding sites on the AuNPs from functioning. The supernatant was discarded after centrifuged at 12,000 rpm at 4 °C for 10 min and also redissolved the precipitate with 100 μL of resuspension buffer (0.01 M PBS pH 7.4, containing 2% *w*/*v* BSA, 1% *w*/*v* sucrose, 0.5% Tween-20, and 0.02% *w*/*v* sodium azide). Finally, the solution was stored at 4 °C [12].

### 2.6. Preparation of Immunochromatographic Strip

The one-step immunochromatographic assay strip operates on the basis of the competitive reaction between the target analyte in the sample and the coating antigen (test line) for the limited colloidal gold-labeled antibody. The sample pad was dripping with 100 µL of each test solution. Afterwards, the specific colloidal AuNPs-labeled IgY antibody was dissolved and migrated along the nitrocellulose membrane through capillary action. While the entire mixture was passing through the membrane, the AuNPs-labeled IgY complex that was solubilized by the sample solution was permitted to react with ciprofloxacin. The colloidal gold-labeled IgY would be captured by the antigen (ciprofloxacin) deposited on the NC membrane if there was no ciprofloxacin in the sample solution. After 20 min, the test result was visually assessed. Positive samples exhibited a single red line (control line), while faint positive samples exhibited two red lines (slight test line and distinct control line). The test was classified as invalid if no control line was present.

**Figure 1:**
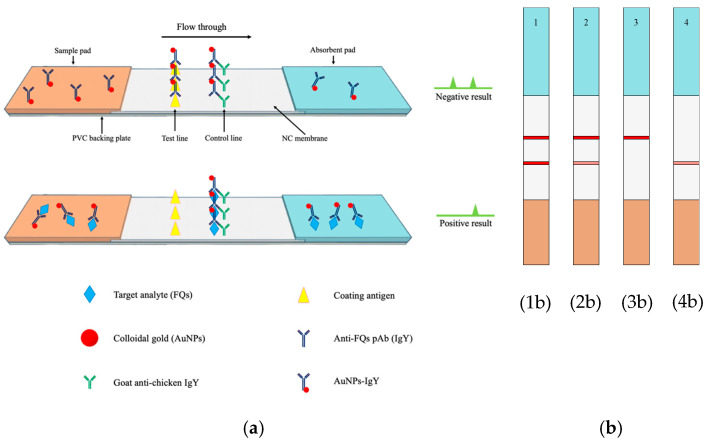
Schematic diagram of the LFIA for detecting FQs by using IgY antibody. (**a**) The structure of LFIA test strip, (**b**) LFIA scheme test result, (**1b**) negative result, (**2b**) weak positive result, (**3b**) positive result, and (**4b**) invalid result.

The test strip comprised four primary components: a polyvinyl chloride (PVC) backing plate as the bottom layer and a nitrocellulose (NC) membrane attached to the middle layer. Moreover, a sample pad and absorbent pad were affixed to the lower and upper sides of the PVC pad, respectively, with an overlap on top of the NC membrane of roughly 2 mm. This method was modified as indicated in the bibliography [12,13]. The diagram of the lateral flow immunoassay (LFIA) is shown in Figure 1. The test line (T line) and control line (C line) were located on the NC membrane before testing. The coating antigens (Cip-OVA, Cip-HSA, and Cip-LF) and the goat anti-chicken IgY were sprayed as the T line and C line, respectively, and diluted with phosphate buffer (PB, 0.1 M, pH 7.4). Furthermore, the NC membrane was dried for at least 4 h at 45 °C and then cut into individual pads with a width of 3 mm. Finally, the strip was stored at room temperature in a hermetically sealed bag containing desiccant material.

The AuNPs-IgY solution was combined with 100 μL of sample extraction buffer solution, thoroughly mixed with a pipette, and incubated for 10 min at room temperature, as depicted in the diagram. Then, 100 μL of the solution mixture was added to the well of the strip test. The mixture was transported from the sample pad to the absorbent pad. This transfer occurred due to the interaction between the antigen coated on the T line and the goat anti-chicken IgY on the C line, which is basically the principle of the antibody–antigen reaction. Subsequently, the detection results were visible to the naked eye after 20 min.

### 2.7. Sample Pretreatment

The primary aim of the present study was to develop an immunochromatographic strip using an IgY polyclonal antibody to become a probe for detecting Cip antibiotics in raw pork meat. The pork samples were collected and analyzed using the developed strips and previous adaptable extraction method [14]. We added 3 g of pork samples into each 15 mL centrifuge tube, containing EDTA and 70% methanol. The mixture was vortexed, agitated, then centrifuged for 5 min. We diluted the extraction solution by transferring 500 µL into 2 mL of ultrapure type I water, resulting in a final concentration of 14% *v*/*v* methanol. The redissolved solution was kept at 4 °C for later analysis.

### 2.8. Sensitivity

The calibration curve was established by utilizing a concentration range of Cip in pork and chicken samples, which were 0–1000 µg/kg. All samples were examined in triplicate. The gold label detector was used to measure the calibration curve, which was plotted on the *X*-axis with the concentration of Cip and the *Y*-axis with the T/C value. The limit of detection (LOD) was determined in order to assess the detection sensitivity. A four-parameter logistic equation was employed to analyze the immunoassay data through curve fitting and statistical analysis. The Prism Pad software version 10 (Prism Pad 10, San Diego, CA, USA) was employed to conduct the calculations.

## 3. Results

### 3.1. Characterization of Immunogen and Coating Antigen (Hapten Density)

**Figure 2:**
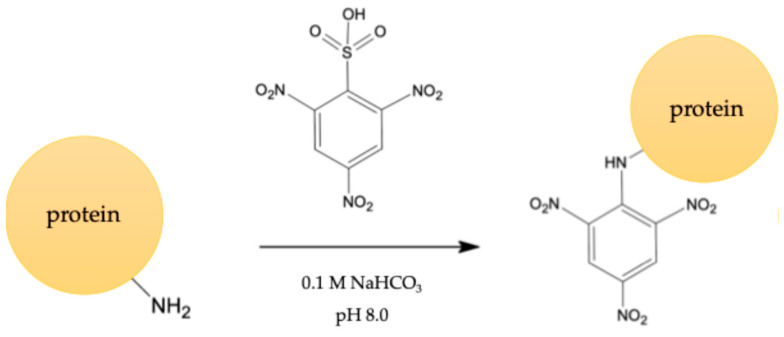
Indirect method of measuring hapten density conjugates using TNBS assay.

The evaluation of the hapten density test was used to evaluate immunogens and coating antigens by a trinitrobenzene sulfonic acid (TNBS) test [15,16]. The number of haptens covalently attached to the surface of a carrier molecule is essential for vaccine efficacy. Figure 2 explains the reaction for quantifying amines in proteins. All samples were purified by dialysis before testing a hapten density. Table 1 shows that the number of amines for BSA-Cip and OVA-Cip conjugates was 3.14 and 2.23, respectively.

### 3.2. Activity of IgY Polyclonal Antibody by Indirect Competitive ELISA

After the fourth injection of immunization in the chicken hen, the IgY polyclonal antibody was collected and purified with the PEG8000 method from the previous study. An IgY consists of two heavy (H) and two light (L) polypeptide chains, arranged in a distinctive Y-shaped structure, including two identical antigen-binding sites similar to mammalian IgG. The structure of the IgY molecule is similar to that of IgG, consisting of two heavy chains (H) with a molecular weight of 67 to 70 kDa each and two light chains (L) with a molecular weight of 25 kDa. Similar to IgG, the light chains contain a constant region (CL) and a variable region (VL). However, IgY has a molecular weight of 180 kDa, while IgG has a molecular weight of 150 kDa [9].

An IgY antibody was developed with indirect competitive ELISA (icELISA), which optimized several parameters. The suitable concentration of the coating antigen (Cip-OVA) and antibody was 20 µg/mL and 1:1000 (final 10 µg/mL), respectively. Moreover, Table 2 illustrates the analysis of the half-maximal inhibitory concentration (IC_50_) using the standard inhibition curve for FQs under this developed ELISA condition.

The specificity of the IgY antibody was assessed through competitive assays against three fluoroquinolone compounds, and the IC_50_ was employed to determine the cross-reactivity percent. Cip exhibits a greater percentage of cross-reactivity with Enro than with itself, as Cip is a major metabolite of Enro. In fact, Enro is optimized explicitly for veterinary applications and frequently has a broader or more potent spectrum of activity against infections typically found in animals.

### 3.3. Characterization of AuNPs and AuNPs-Labeled IgY pAb

**Figure 3:**
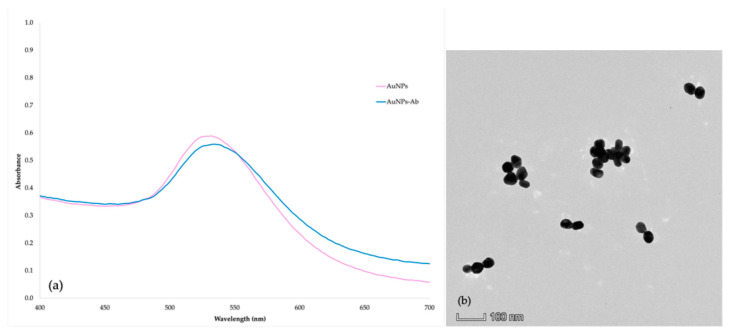
The characteristics of AuNPs and AuNPs-labeled IgY pAb: (**a**) the UV-visible spectrum graph of AuNPs and AuNPs-labeled IgY pAb and (**b**) transmission electron microscopy image of AuNPs.

The purified IgY antibody proved completely pure and intact. The anti-FQs IgY concentration was 11 mg/mL. The titer of the antibody as determined by ELISA was as elevated as 1:128,000. The analysis of the morphology and dimensions of the AuNPs is significant in assessing the stability of colloidal gold. To ensure the effective conjugation of the antibody and the colloidal gold, the AuNPs–antibody complexes were analyzed using UV-visible spectroscopy. A red shift in the maximum absorption wavelength was observed compared to the bare colloidal gold nanoparticles. An antibody protein surrounding the tagged gold particles was also detected. UV-visible spectroscopy was employed to scan the AuNPs within the 400 to 700 nm wavelength range. A single peak of maximal absorbance was obtained at a wavelength of 534 and 532 nm for AuNPs and AuNPs-labeled IgY pAb, respectively (Figure 3a). In addition, the average particle size of AuNPs was less than 100 nm, as indicated by the transmission electron microscopy (TEM) characterization in Figure 3b. The interaction between AuNPs and antibodies was typically attributed to electrostatic attraction. Under specific circumstances, AuNPs have a negative surface charge, while antibodies possess a positive surface charge [12].

### 3.4. Optimization of LFIA Strip

#### 3.4.1. The Labeling pH Value

**Figure 4:**
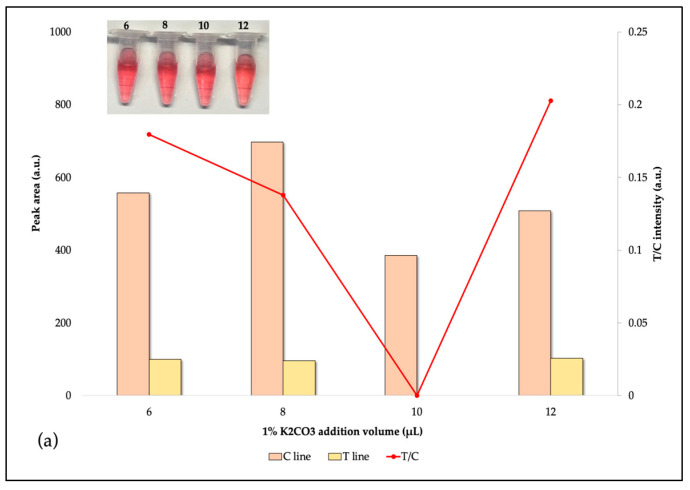

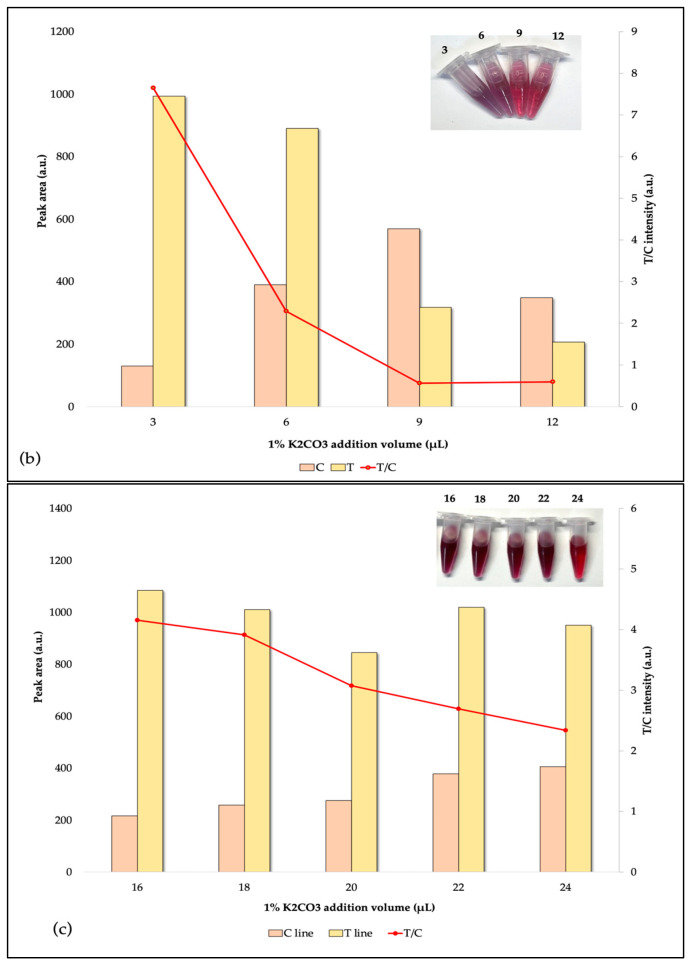
The effect of pH value and AuNPs concentration on IgY antibody-labeled AuNPs: (**a**) 1:10,000 of AuNPs concentration, (**b**) 2:10,000 of AuNPs concentration, and (**c**) 4:10,000 of AuNPs concentration.

The pH reaction levels were crucial to the effectiveness of AuNPs–Abs conjugates. Theoretically, the pH of the reaction is supposed to be moderately elevated over the isoelectric point of protein. Below the isoelectric point, antibodies may clump together, causing AuNPs–Abs to clump together and precipitate. This would lower the accuracy and cause false negatives. Above the isoelectric point, the adsorption action is limited by charge repulsion between the AuNPs and the antibodies. This makes the test strip lighter in color. Based on signal intensity and sensitivity (Figure 4), the best pH for the AuNPs solutions was 7.8, which was reached by adding 9 µL of 0.1 M K_2_CO_3_ solution. The dimensions and concentration of AuNPs also influence the interaction between the antibody and the target analyte under investigation. According to the findings of the tests, the suitable concentration of AuNPs was 2:10,000.

#### 3.4.2. Concentration of Coating Antigen or Test Line (T-Line) and IgY Antibody

The optimal concentrations of the AuNPs-labeled IgY antibody and coating antigen were established through the chessboard titration method. The results indicated that the most effective concentrations for the AuNPs-labeled antibody and coating antigen were 1.25 μg/mL and 0.50 mg/mL, respectively.

**Figure 5:**
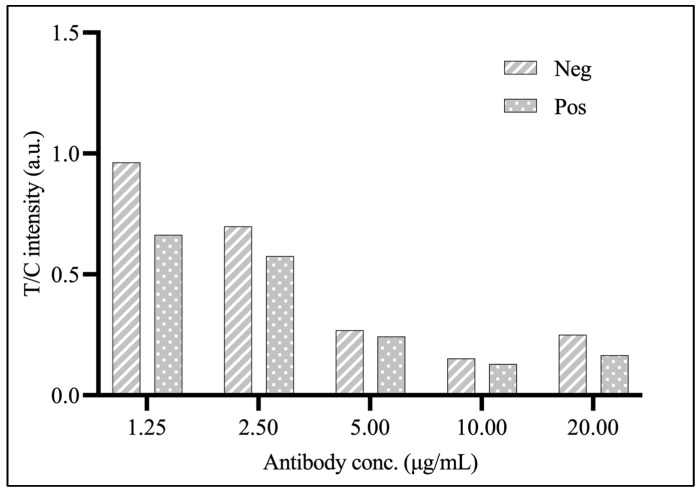
The optimization of antibody concentration.

The results shown in Table 3 demonstrate that the suppression of the antibody to the test substance is less than 1.00 percent inhibition when coated antigen concentrations are more than 0.50 mg/mL. Therefore, coating antigen concentrations of 0.5 and 0.25 are appropriate for the reaction on the strip test. In addition, it is worth noting that the optimal antibody concentration for usage as a probe is 1.25 µg/mL, which shows the most significant difference in T/C values in negative and positive results, as shown in Figure 5.

#### 3.4.3. Concentration of Secondary Antibody or Control Line (C-Line)

**Figure 6:**
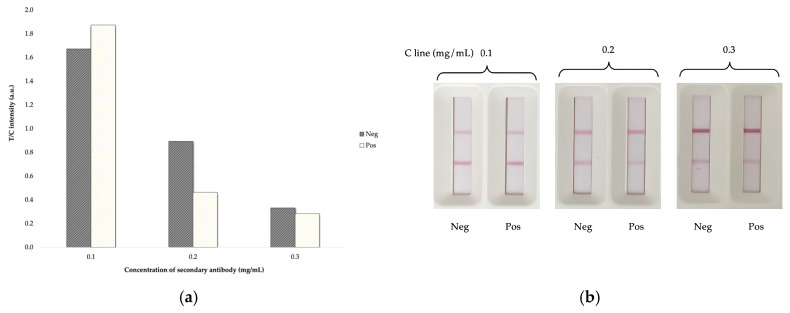
The optimization of secondary antibody concentration (C line) for LFIA strip: (**a**) the T/C response value and (**b**) the LFIA test strip image.

For the LFIA analysis of FQs in the sample, a test was needed to make sure that the antigen and antibody were able to bind by using AuNPs-IgY probes to catch anti-IgY that are in the C-line. To confirm the authenticity and suitability of the strip test reactivity, we coated the control line with anti-chicken IgY. According to the results, the secondary antibody that is most appropriate is 0.2 mg/mL, which demonstrates the most significant disparity between the negative result and the positive result (Figure 6).

#### 3.4.4. Buffer Solution of LFIA Strip

**Figure 7:**
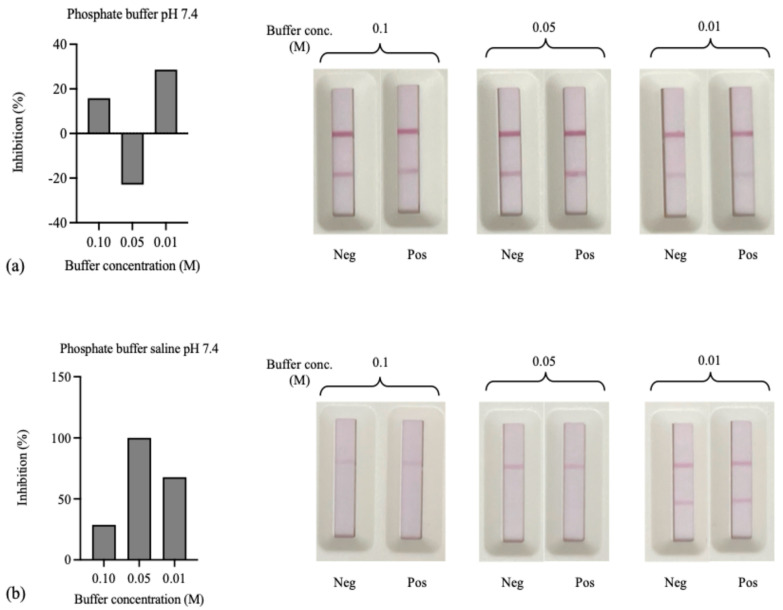

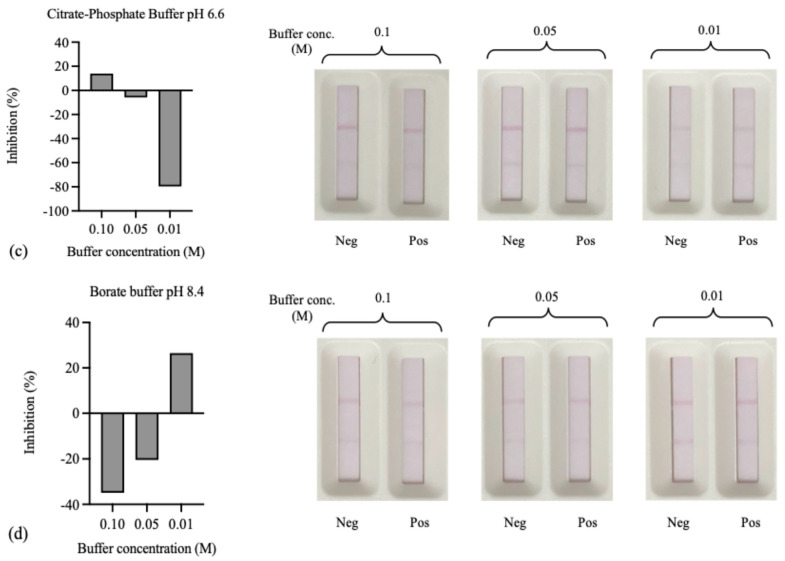
The optimization results of the buffer solution dissolved AuNPs-IgY probe for the LFIA test strip: (**a**) phosphate buffer pH 7.4, (**b**) phosphate buffer saline pH 7.4, (**c**) citrate–phosphate buffer pH 6.6, and (**d**) borate buffer pH 8.4.

This study revealed that selecting a resuspension buffer affects the stability of AuNPs–IgY antibody conjugates. We evaluated multiple buffers, including phosphate buffer (pH 7.4), phosphate buffer saline (pH 7.4), borate buffer solutions (pH 8.0), and citrate–phosphate buffer (pH 6.6) for 0.1, 0.05, and 0.01 M in each buffer to assess their impact on conjugate stability and assay sensitivity. The results showed that formulating the AuNPs–Ab conjugate with an antibody concentration of 1 µg/mL achieved optimal sensitivity for the immunochromatographic strip test, as shown in Figure 7. The AuNPs–IgY antibody conjugates had the highest inhibition rate on the strip test when PB was used as a resuspension buffer. This means they were most stable. The 0.01 M phosphate buffer solution at pH 7.4 exhibited superior stability and sensitivity for the AuNPs–Abs conjugates. We selected this buffer as the resuspension solution to prevent coagulation and maintain test efficacy.

#### 3.4.5. Enhancement of Buffer Solution for T-Line

Table 4 shows the results obtained from assessing the type of solution contained in the buffer. A sample suitable for LFIA analysis is needed to increase the color intensity of the T-line in the immunochromatographic strip assay. Several buffer additives were tested, including BSA, skim milk, Tween-20 (T-20), PEG6000, PVP, and sucrose, as listed in Table 2. The results demonstrated that a buffer containing 1% of Tween-20 was the most effective in enhancing the color intensity of the T-line, resulting in more comprehensible results. Therefore, we selected this buffer composition as the best way to maximize color development on the strip.

#### 3.4.6. Methanol Effect

The investigation of the extraction of Cip using the previous method [11] determined that methanol was used as a component of the fluoroquinolone antibiotic extraction from meat samples. In this study, we investigated the effect of methanol concentration on the stability and performance of the AuNPs-IgY antibody probe in the immunochromatographic strip assay. Methanol concentrations ranging from 0% to 20% were found to have no observable impact on the stability or effectiveness of the AuNPs-IgY probe, as indicated by consistent T/C values across these concentrations.

**Figure 8:**
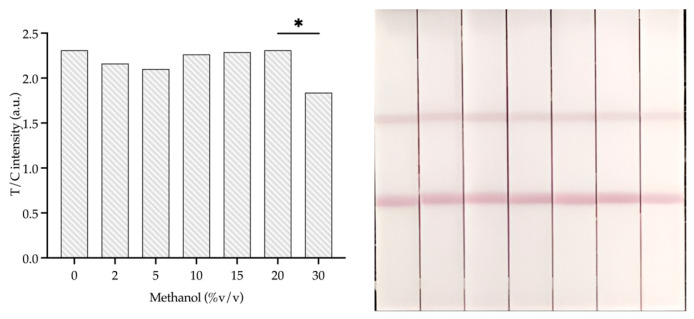
The methanol percentage affects the signal measurement T/C value. * Mean *p*-value correspondingly compared with 20% and 30% *v*/*v* of methanol by *t*-test, is significantly different (*p* < 0.001).

However, Figure 8 shows a significant (*p* < 0.001) decrease in the T/C value when the methanol concentration reached 30%. This means that when the amount of methanol increases, it might start to get in the way of the binding interactions between the AuNP-IgY probe and the target, or it might make the colloidal gold–antibody conjugate a little less stable. Therefore, we should ideally keep methanol concentrations at or below 20% for the optimal performance and stability of the AuNPs-IgY antibody probe.

#### 3.4.7. Specificity and Sensitivity

**Figure 9:**
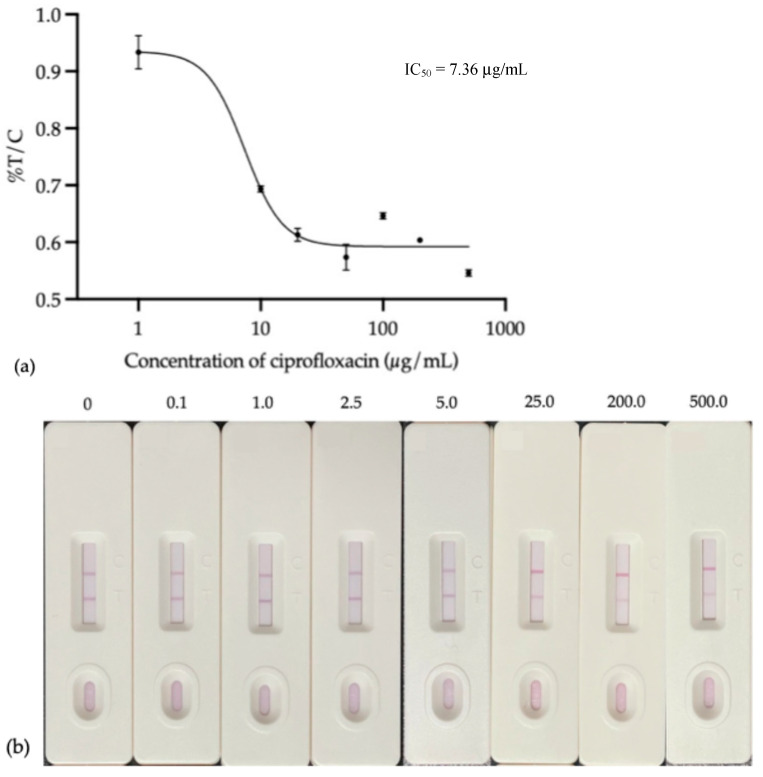
Inhibition curve of ciprofloxacin by LFIA strip test: (**a**) standard curve and (**b**) inhibition strip test.

We assessed the sensitivity and specificity of the immunochromatographic strip assay and found the IC_50_ value in Figure 9, the amount of analyte that blocks the signal by 50% was 7.36 µg/mL. This IC value indicates the assay’s sensitivity, revealing the concentration at which competition with the target analyte significantly reduces the test line signal.

The assay’s ability to find the target analyte even when other substances were present with little cross-reactivity proved that it was specific. The sensitivity of the developed strip test using the AuNPs-IgY antibody was assessed by analyzing a range of ciprofloxacin concentrations shown in Figure 8. Moreover, the developed strip test could be detected during a range of inhibitory concentrations, from IC_90_ to IC_2.5_, as identified by the limits of detection (LOD), which were 16.05 and 0.20 µg/mL, respectively.

#### 3.4.8. Matrix Effect

**Figure 10:**
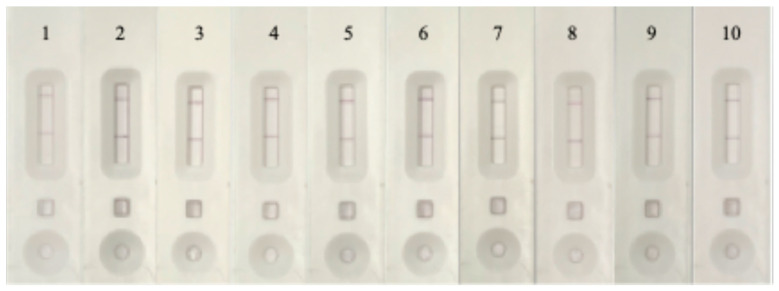
The correlation graph of matrix extraction from sample affected to LFIA test strip by 1–10: 0, 1, 5, 10, 20, 50, 100, 200, 300, and 500 µg/mL analyzed by LFIA test strip; in pork matrix.

The matrix effect was assessed by using extracts from chicken and hog samples to test the immunochromatographic strip assay. The assay’s signal significantly decreased, according to the results by spiked standard ciprofloxacin shown in Figure 10, suggesting that some of the ingredients in the pork extracts are interfering with the assay’s functionality. This matrix effect is likely caused by chemicals in the meat extracts that either interact with the AuNPs-IgY antibody probe or stop the antibody from binding to the target analyte. In complicated sample matrices, these matrix interferences might lower the assay’s sensitivity and accuracy in identifying the target analyte. Additional sample preparation procedures, including dilution, filtration, or a buffer designed for complex matrices, would be necessary to lessen these effects and enhance test reliability when used on chicken and pork samples.

#### 3.4.9. Comparison of the Multiplex LFIA Strip Test

Table 5 summarizes the results of the multiplex LFIA strip tests for fluoroquinolone in recent years. The detection targets of the published papers were fluoroquinolone antibiotics used in food products. The published LFIAs primarily used qualitative or semi-quantitative detection methods. The table provides comparative data for different methods and probes for the determination of ciprofloxacin.

To clarify, the developed immunoassays are proficient in identifying ciprofloxacin antibiotic residues. However, the sensitivity and efficacy of specific immunoassays vary from conventional LFIA using mAb probe methods, depending on the matrix effect present in the extracted solution. Furthermore, the particular immunoassay techniques demonstrated significant sensitivity, suggesting that the immunoassay method is comparable to the conventional LFIA method.

## 4. Discussion

Ciprofloxacin antibiotics are essential for livestock production and are also used to treat diseases caused by bacteria. Previously, several ELISA methods [23,24] have been developed to detect antibiotic residues. Although this technique is sensitive, the test must be performed by professionals, is time consuming, and requires special equipment, such as microplate readers, which are unsuitable for field use. Therefore, extensive research is underway to develop new, rapid, and convenient methods for analyzing this substance by the LFIA method [25,26]. Still, such methods require the production of specific antibodies for analysis, which are expensive and complicated.

This work effectively developed a lateral flow immunoassay strip utilizing gold nanoparticles coated with egg yolk antibodies (IgY) to detect ciprofloxacin antibiotics in pork and chicken samples. Ciprofloxacin is a major metabolite of enrofloxacin, and it is optimized for human bacterial treatment. We prioritized ciprofloxacin residue over enrofloxacin because if ciprofloxacin residues are found in meat, they will accumulate in the long term and eventually lead to drug resistance, which will have serious direct impacts on human health [27].

The LFIA requires no trained personnel or specialized tools, and results can be obtained within 20 min. This technique is cost effective as it necessitates only a minimal quantity of immunoassay reagents. The success of the LFIA is largely dependent on the quality of the labeling antibody. An anti-FQs IgY pAb was used instead of mAb or alternative antibodies which have easy production. The yolk of chicken eggs contains a molecule akin to immunoglobulin (Ig), referred to as IgY. Chicken IgY offers considerable benefits over mammalian IgG. Furthermore, chicken IgY identifies a greater number of epitopes and possesses an expanded antibody repertoire, leading to signal amplification. The utilization of IgY will diminish cross-reactivity and background due to its evolutionary divergence. Thus, false positives are eliminated in immunological experiments, a concern associated with IgG-based mammalian antibodies [28,29]. The stability of IgY during storage is relatively high. IgY solutions could be preserved at 37 °C for one month, at 4 °C for several months, and for several years without substantial degradation of antibody activity [30].

The antibody sticks to the gold solution better when the pH slightly exceeds the labeling antibody’s isoelectric point (pI); the pI of IgY is 5.2. The purity of the antigen and the pH of the gold solution correlate with the sensitivity and specificity of the LFIA tests [31,32].

Nevertheless, although IgY exhibits exceptional specificity for antibiotics, it is not enough to provide efficacy compared to mAb, which is a limitation of our study. So, it is imperative to evaluate additional criteria to facilitate the substitution of other antibodies with the IgY antibody while guaranteeing high efficacy.

## 5. Conclusions

This study developed and optimized a one-step lateral flow immunoassay strip test for detecting ciprofloxacin, achieving effective sensitivity and specificity under optimized conditions: pH was 7.8 and the buffer composition was 20% methanol containing 1%T20 in 0.01 M phosphate buffer pH 7.4. Antibody and antigen concentrations were 1.25 µg/mL and 0.50 mg/mL, respectively. The secondary antibody was 0.2 mg/mL, while identifying a moderate IC_50_ of 7.36 µg/mL and a limit of detection was 0.2 µg/mL, suggesting the need for additional sample preparation to enhance the reliability in food safety applications.

## Figures and Tables

**Table 1:** The hapten density of coating antigen and immunogen.

Haptens *	Hapten Density (Number of Amines/Protein)
BSA-Cip (immunogen)	3.14
OVA-Cip (coating antigen)	2.23

* Prepared hapten of 890 µg/mL in 0.1 M sodium bicarbonate pH 8.0. The solution was reacted with 0.01% TNBS, and it was measured at 420 nm using a spectrophotometer.

**Table 2:** The cross-reactivity percent of IgY antibody.

FQs	IC_50_ (µg/mL)	Cross-Reactivity Percent
Ciprofloxacin	0.82	100.00
Enrofloxacin	0.37	218.22
Norfloxacin	2.06	39.76

**Table 3:** Coating antigen concentration and inhibition of ciprofloxacin on strip test.

Coating Antigen (mg/mL)	T/C Intensity (a.u.)		Percentage Inhibition (%)
	Negative	Positive	
5.00	1.2657	1.4154	<1.00
2.00	1.1101	1.4895	<1.00
1.00	0.6018	0.7850	<1.00
0.50	0.9636	0.6642	31.07
0.25	1.0515	0.7150	32.01

**Table 4:** Assessment of the buffer solution for enhancing the color level of the strip, comprising BSA, skim milk, T-20, PEG6000, PVP, and sucrose.

Solution	Concentration (% *v*/*v*)	T/C		Inhibition (%)
		Negative	Positive	
BSA	2.0	0.2230	0.1466	34.25
	1.0	0.2786	0.2332	16.28
	0.5	0.2806	0.4778	<0.01
Skim milk	2.0	0.9320	0.5250	43.67
	1.0	0.7280	0.5474	24.80
	0.5	0.8155	0.7104	12.89
Tween-20	2.0	0.4930	0.5413	<0.01
	1.0	0.7436	0.3997	46.25
	0.5	0.6436	0.3713	42.31
PEG 6000	2.0	0.4244	0.2984	29.70
	1.0	0.4134	0.2687	34.99
	0.5	0.2125	0.2840	<0.01
Polyvinylpyrrolidone	2.0	0.7509	0.5596	25.47
	1.0	0.3787	0.4293	<0.01
	0.5	0.4868	0.3442	29.28
Sucrose	2.0	0.3734	0.3773	<0.01
	1.0	0.2045	0.5419	<0.01
	0.5	0.5010	0.3733	25.48

**Table 5:** Comparison of fluoroquinolones multiplex LFIAs.

Detection Methods	Target Analytes	Samples	Type of Antibody	LOD	References
AuNPs-LFIA	Enro, Cip, Flu, Mar, Oxo, Dano	blood and meat	IgG mAb	50, 50, 100, 75, 50, 50 µg/kg	[17]
Au@Ag NPs-LFIA	Gat	meat	IgG mAb	0.8 pg/mL	[18]
AuNPs-LFIA	Enro	milk	IgG mAb	0.078 ng/mL	[19]
MNPs-LFIA	Cip, Dan, Dif, Enro, Lom, Mar, Nor, Ofl, Orb, Sar	milk, raw milk	IgG mAb	1–2 ng/mL	[20]
AuNPs-LFIA	Cip, Lev	milk	IgG pAb	0.05, 0.1 ng/mL	[21]
EuNPs-LFIA	FQs	honey, milk, chicken, egg	IgG mAb	2.4 ng/mL	[22]
AuNPs-LFIA	Cip	raw pork	IgY pAb	0.2 µg/mL	This work

mAb = Monoclonal antibody, Enro = Enrofloxacin, Cip = Ciprofloxacin, Flu = Flumequine, Mar = Marbofloxacin, Oxo = Oxolinic acid, Dano = Danofloxacin, Gat = Gatifloxacin, Dan = Danofloxacin, Dif = Difloxacin, Lom = Lomefloxacin, Nor = Norfloxacin, Ofl = Ofloxacin, Orb = Orbifloxacin, Sar = Sarafloxacin, Lev = Levofloxacin, FQs = Fluoroquinolones, AuNPs-LFIA = Lateral flow immunoassay based on gold nanoparticle, Au@Ag NPs-LFIA = Bimetallic nanoparticles consisting of a gold core and a silver shell, MNPs-LFIA = Magnetic nanoparticle-based lateral flow immunochromatography assay, EuNPs-LFIA = Fluorescent immunoassay based on europium nanoparticles.

## Data Availability

The original contributions presented in this study are included in the article. Further inquiries can be directed to the corresponding authors.

## Abbreviations

The following abbreviations are used in this manuscript:

LFIA	lateral flow immunoassay
AuNPs	gold nanoparticle
Cip	ciprofloxacin
pAb	polyclonal antibody
mAb	monoclonal antibody
T line	test line
C line	control line
IC_50_	half-maximal inhibitory concentration
IC_10_	inhibitory concentration at 10%
IC_90_	inhibitory concentration at 90%.
